# Papillary Endolymphatic Sac Tumor: A Case Report

**DOI:** 10.1155/2012/163851

**Published:** 2012-07-17

**Authors:** S. Arava, R. M. Soumya, S. Chitragar, R. Safaya, S. H. Chandrashekhar, Alok Thakar

**Affiliations:** ^1^Department of Pathology, All India Institute of Medical Sciences, Academic Building, Ansari Nagar, New Delhi 110 029, India; ^2^Department of Radio Diagnosis, All India Institute of Medical Sciences, Academic Building, Ansari Nagar, New Delhi 110 029, India; ^3^Department of Otorhinolaryngology, All India Institute of Medical Sciences, Academic Building, Ansari Nagar, New Delhi 110 029, India

## Abstract

Glandular tumors involving the middle ear are rare and distinguishing between adenoma and adenocarcinoma remains difficult. A distinct subclass of these tumors demonstrates microscopic papillary architecture and has a propensity to erode the petrous bone and extend intracranially. The term “aggressive papillary middle ear tumor” has recently been proposed to describe this more invasive type of middle ear tumor. These tumors cause symptoms even when microscopic in size. Although histologically benign, they have been locally destructive with frequent intracranial extension and patients may die of uncontrolled local disease. These tumors do not metastasize but there is single case report of drop metastasis to the spine in the literature. Hence this tumor must be distinguished from other benign tumors of the middle ear. These rare neoplasms constitute a distinct pathological entity and deserve wider recognition.

## 1. Introduction

Endolymphatic sac tumors are rare, and their true origin is not clear [1]. Although histologically benign, they may exhibit invasive growth and destruction of the skull base [2]. In the past, a middle-ear origin was presumed. Only recently convincing evidence exists that these tumors in fact arise from the Endolymphatic sac [3]. Patients may present with various symptoms and signs including tinnitus, hearing loss, vertigo, and even facial paralysis [4]. Generally histologically benign in appearance, they have been misdiagnosed as middle ear adenomas, adenocarcinomas, or choroid plexus papilloma's [2, 5]. A few cases have been associated with von Hippel-Lindau disease [6]. Here we report a rare case of papillary endolymphatic sac tumor with clinical, radiological, histological, and immunohistochemical findings.

## 2. Case Summary

**Figure 1:**
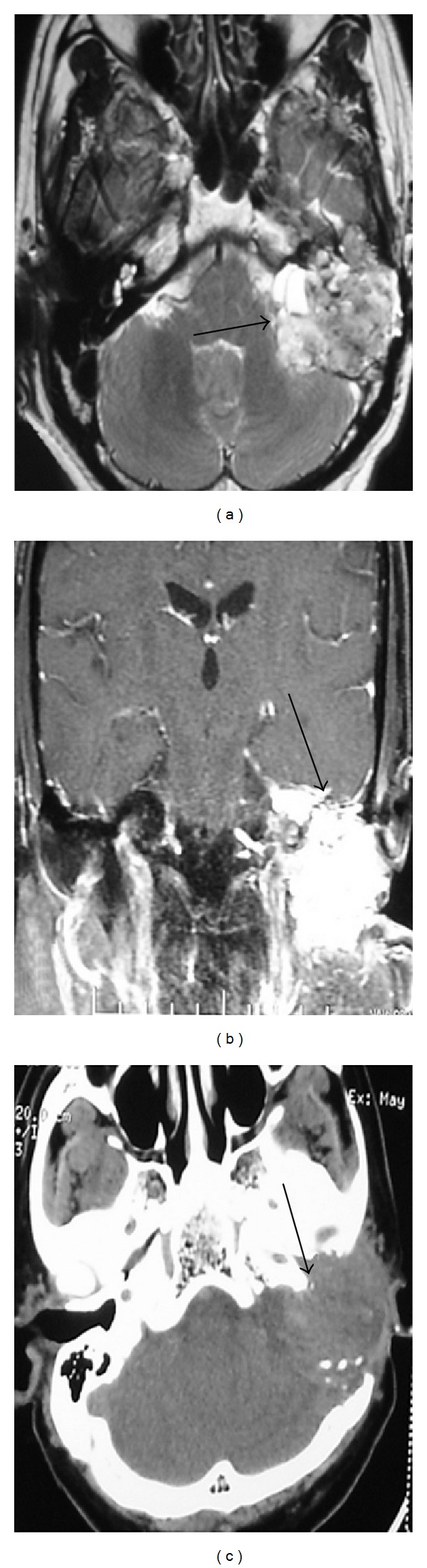
Axial MRI T2-weighted image (a) and contrast-enhanced coronal T-weighted image (b) showing heterogenous intensely enhancing mass involving left petrous temporal bone causing destruction of bone, extending medially left cerebellopontine angle cistern and inferiorly below base of skull. Axial CT scan (c) showing destruction of extensive destruction of the posterior petrous bone and middle ear structures.

**Figure 2:**
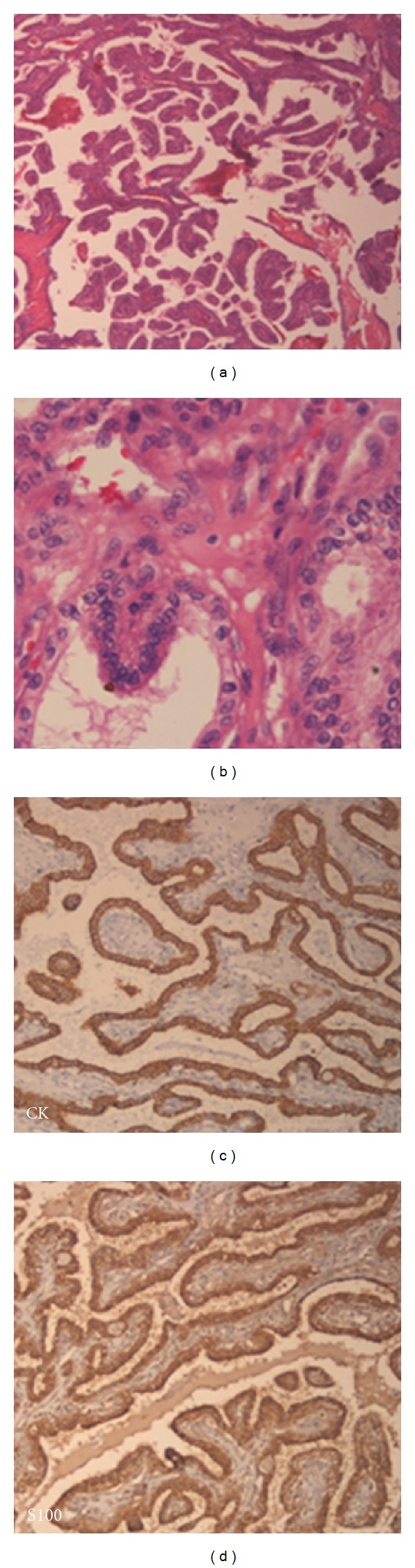
Microscopy shows typical papillary structures (2(a), H&E × 100), lined by benign looking low cuboidal epithelial cells (2(b), H&E × 200). Tumor cells are immunopositive for cytokeratin (2(c), IHC (CK)[Daco] × 200) and S100 (2(d), IHC (S100)[Daco] × 200).

63-year-old male patient came with the history of otalgia, hearing loss, lower cranial nerve palsy, and recurrent bleeding in the left ear since 6 years. On examination a reddish blue mass bulging from the tympanic membrane was seen in the left ear. MRI demonstrated (Figure 1(a), arrow) a large heterogenous mass involving left petrous temporal bone causing destruction of bone (Figure 1(b), arrow), extending medially left cerebello-pontine angle cistern and inferiorly below base of skull. The tumor exhibited heterogeneous signal intensity on both T1- and T2WI and strong homogeneous enhancement. Computed tomography (CT) showed extensive destruction of the posterior petrous bone and middle ear structures with involvement of internal auditory canal (Figure 1(c), arrow). The preoperative diagnosis was Glomus jugulare tumor. Patient underwent mastoid, exploration by Type A lateral skull base approach with Type IV thyroplasty and left upper eye lid gold weight implant. On exploration, middle ear, mastoid and jugular foramen was involved by the tumor. Complete removal of the tumor were done along with preservation of IX (accessory) and XII (hypoglossal) cranial nerves but left facial nerve is sacrificed. Postoperatively there was no locoregional recurrence, wound was healthy and voice is improved. Microscopically (Figures 2(a) and 2(b)) the tumor was composed of typical papillary structures with central fibrovascular core lined by benign looking, single layer of cuboidal to columnar epithelial cells. Nuclear pleomorphism and mitotic activity are not seen. The cytoplasm was moderate to abundant and eosinophilic. Immunohistochemically the tumor cells are positive for cytokeratin (CK), S100 (Figures 2(c) and 2(d)) and epithelial membrane antigen (EMA). They are negative for thyroid transcription factor (TTF-1), neuron-specific enolase (NSE), chromogranin (CG), synaptophysin (SYN), and glial fibrillary acidic protein (GFAP).

## 3. Discussion

Tumors with papillary architecture arising from the epithelium of the middle ear and invading into the adjacent bony structures has been called as “Primary aggressive papillary tumor of the middle ear” by Gaffey et al. [7]. Heffner in his study of 20 cases postulated that these tumors arise from the complex rugose portion of the endolymphatic sac, which extends from its intraosseous origin to its intradural extra osseous terminus [6]. The tumor affects adults of both sexes with age range from 17 to 71 years. The clinical prodrome is prolonged. Most common symptoms include hearing loss, otalgia, tinnitus, vertigo, and facial weakness [8]. On examinations reveal red or blue tumor just behind the tympanic membrane. Radiologically these tumors are hypervascular as evidenced by angiography. Although these tumors do not metastasize, in a recent case report, Bambakidis et al. showed that it can metastasize to distant sites [2]. endolymphatic sac tumors were established as a part of the VHL syndrome in 1997 by Manski and colleagues, which is an autosomal dominant disorder associated with both malignant and benign neoplasms including hemangioblastomas of the cerebellum, retinal angiomas, pancreatic cysts, and pheochromocytomas. These tumors tend to be bilateral when associated with VHL syndrome, but they can occur rarely in individuals who do not have a mutation or deletion of the VHL gene also [9]. Our patient did not have any other associated abnormality on thorough examination. In most of the previously reported cases including the present one, there was invasion of the portion of the petrous temporal bone, demonstrated as lytic lesion by CT. Although these tumors are histologically benign with rare mitotic figures, they are locally aggressive neoplasms hence radical resection is the treatment of choice although complete resection may not be possible. Recurrence may occur due to subtotal resection [2]. The differential diagnosis of endolymphatic sac tumor includes other destructive lesions of the temporal bone such as paraganglioma, meningioma, hemangiopericytoma, and metastases [10]. Radiologically hypervascular mass near the temporal bone is strongly suggestive of a paraganglioma. Typical Zellballen pattern along with immunopositive for chromogranin and synaptophysin distinguishes it from Endolymphatic sac tumor. Rare cases of papillary meningioma have been reported in the temporal bone but they are cytologically anaplastic with areas of necrosis, pleomorphism, and high mitotic activity. Metastatic lesions to the temporal bone may cause difficulty in diagnosis but proper work up along with immunohistochemical stains will help to distinguish between the two.

## 4. Conclusion

Endolymphatic sac tumors are rare skull base tumor originates from endolymphatic epithelium within the vestibular aqueduct, characterized clinically by slow growth with local invasion and bone destruction. Metastasis is reported in only one case. Histologically it has bland cytologic features and a papillary growth pattern. Surgical exploration of the tumor and sac is the treatment of choice but recurrence may occur due to subtotal resection.
